# Supplementary material for: Association between oral microbiome and seven types of cancers in East Asian population: a two-sample Mendelian randomization analysis
Source: Front Mol Biosci. 2023 Nov 21;10:1327893. doi: 10.3389/fmolb.2023.1327893 (PMC10702768; doi:10.3389/fmolb.2023.1327893)
Supplement: Supplementary file 1 [file DataSheet1.ZIP › Supplementary Materials/MR plots of saliva/saliva═╝/ovarian cancer/pheno.2484_to_ovarian cancer_leave_one_out.pdf]

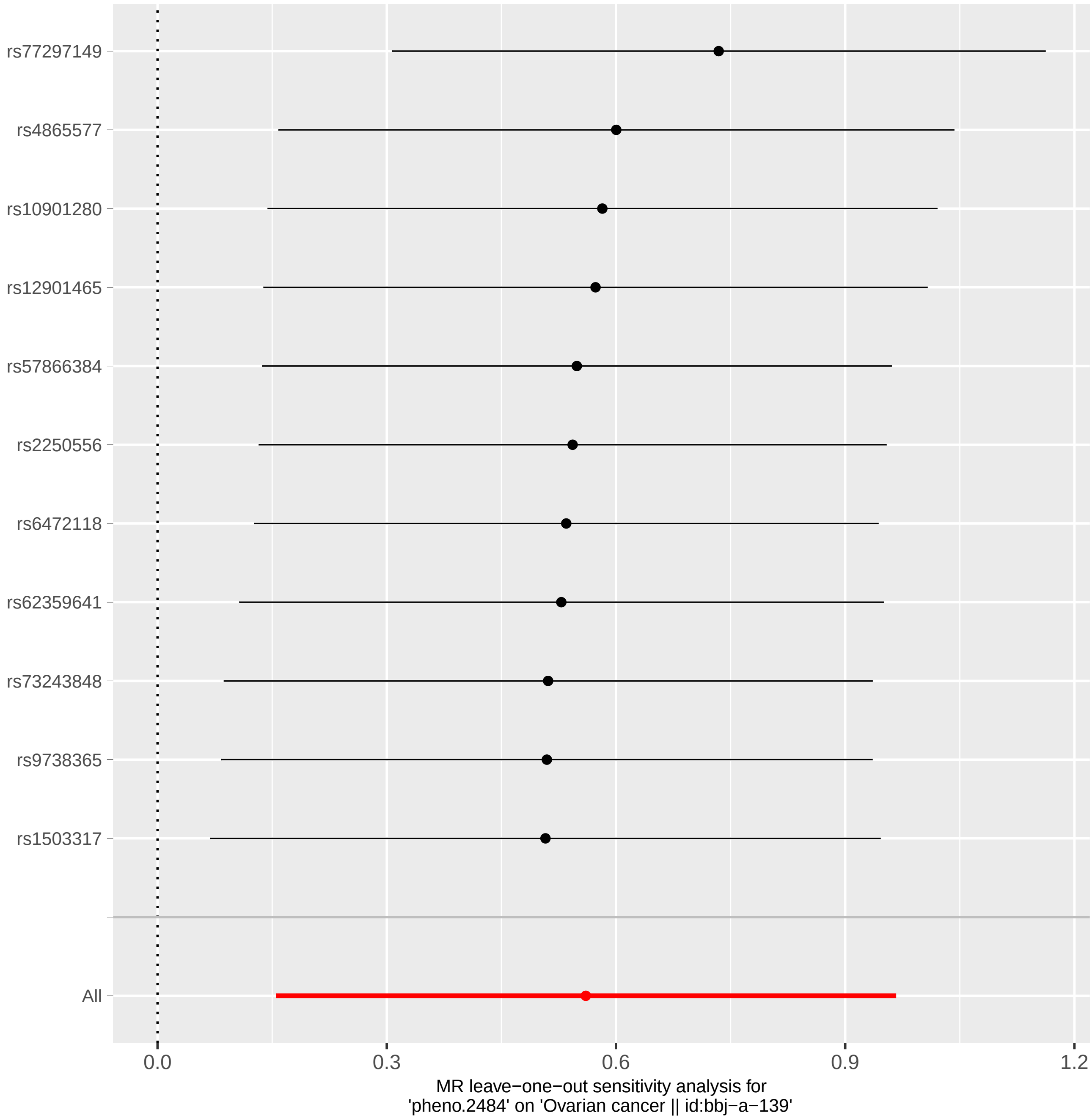
rs77297149
rs4865577
rs10901280
rs12901465
rs57866384
rs2250556
rs6472118
rs62359641
rs73243848
rs9738365
rs1503317
All
0.0
0.3
0.6
0.9
1.2
MR leave−one−out sensitivity analysis for
'pheno.2484' on 'Ovarian cancer || id:bbj−a−139'
